# Le glaucome congénitale et la neurofibromatose type 1

**DOI:** 10.11604/pamj.2015.21.56.6794

**Published:** 2015-05-25

**Authors:** Nazih Tzili, Hamza El Orch, Fatiha Bencherifa, Mohammed Charif Chefchaouni, El Hassan Abdallah, Amina Berraho

**Affiliations:** 1 Service d'Ophtalmologie B, Hôpital des Spécialités, CHU Rabat, Maroc

**Keywords:** Glaucome congénitale, neurofibromatose type 1, étiopathogénique, congenital glaucoma, neurofibromatosis type 1, etiopathogenic

## Abstract

Le glaucome congénital constitue une complication ophtalmologique dans la neurofibromatose type 1 ou maladie de von Recklinghausen. Nous rapportons un cas de glaucome congénitale dans le cadre d'une neurofibromatose de type 1, à travers lequel on va discuter les mécanismes étiopathogénique, les difficultés thérapeutiques et les facteurs pronostiques du glaucome congénital au cours la maladie de von Recklinghausen avec revue de la littérature.

## Introduction

La neurofibromatose de type 1 (NF1) est une phacomatose héréditaire autosomique dominante fréquente, décrite la première fois par Von Recklinghausen en 1882 [1]. Elle se traduit par une dysplasie généralisée neuroectodermique et mésodermique, affectant de façon progressive et polymorphe le derme, le système nerveux, le squelette et le système vasculaire. Le glaucome congénital constitue une complication ophtalmologique rare dans la neurofibromatose type 1 ou maladie de von Recklinghausen et la plupart des publications ophtalmologiques dans la littérature sur le glaucome et la NF1 sont dérivé des rapports de 1 ou 2 cas [2–6]. Nous rapportons un cas de glaucome congénitale dans le cadre d´une neurofibromatose de type 1.

## Patient et observation

**Figure 1:**
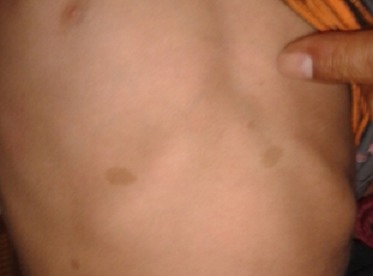
Taches cutanées de couleur café au lait sur le corps de l'enfant

**Figure 2:**
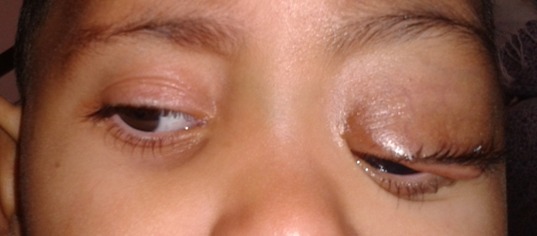
Névrome plexiforme de la paupière supérieure gauche

**Figure 3:**
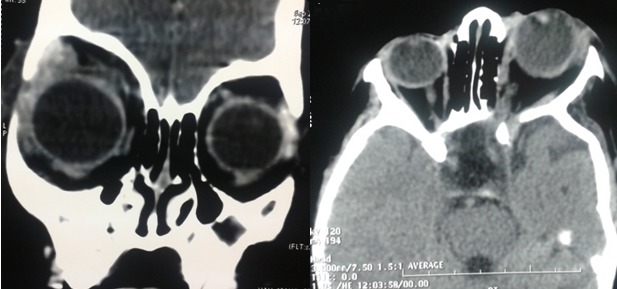
Coupes scannographiques objectivant la buphtalmie et un élargissement du globe oculaire avec élargissement de la cavité orbitaire

Un enfant âgé de 3 ans, se présente avec des taches cutanées de couleur café au lait sur tout le corps (Figure 1) et une hémihypertrophie faciale. Sur le plan oculaire, du côté gauche on retrouve un névrome plexiforme de la paupière supérieure (Figure 2), une buphtalmie, une cornée oedematiée et dystrophique avec un tonus à 24mmhg. On note également la présence de nodules iriens de lish. Le fond de l’œil est difficile à analyser. L'examen de l’œil droit est sans particularité. L'examen neuroradiologique (TDM) objective un globe augmenté de taille avec un élargissement de la cavité orbitaire (Figure 3) et une dysplasie de l'hémiface gauche. Par ailleurs, la TDM ne décèle pas de gliome tout au long des voies visuelles. Une trabéculectomie sous mitomycine a été réalisée. Le pronostic de cet œil chez notre enfant est resté réservé à cause du retard diagnostic et de l'amblyopie sévère avec des examens électro-physiologiques très altérés.

## Discussion

La neurofibromatose de von Recklinghausen est une maladie autosomique dominante à manifestations polyviscérales qui présente des complications ophtalmologiques. Le glaucome congénital constitue une complication ophtalmologique dans la neurofibromatose type 1 ou maladie de van Recklinghausen. Il est généralement uni ou bilatéral, habituellement précoce, de type congénital avec buphtalmie. Notre enfant s'est présenté dans le cadre du syndrome de jules François. Ce syndrome, décrit en 1956 par J. François [7], associe un glaucome congénital précoce unilatéral, un neurofibrome plexiforme de la paupière supérieure homolatérale et une hémihypertrophie de la face. Une dysplasie osseuse et des formations tumorales peuvent s'associer à la triade. Concernant les mécanismes étiopathogéniques, l'infiltration de la gaine de Schwann des nerfs ciliaires (neurofibromes) qui bloquent l'angle est de loin le mécanisme le plus fréquemment cité pour le glaucome associé à la NF1 [2, 3, 8]. Mais d'autres mécanismes ont été envisagés, y compris des malformations ou de développement immature de l´angle de la chambre antérieure [2, 9]. Une combinaison de mécanismes potentiels, y compris les anomalies congénitales, des troubles pigmentaires de l´angle, et fermeture de l´angle secondaire par des synéchies antérieures est aussi envisageable [10]. L'ectropion uvéal a été rapporté chez des patients présentant la NF1 orbito-faciale et uniquement sur le même côté, il est donc préconisé que tout nourrisson ou enfant présentant un ectropion uvéal doit être suivie pour un risque de développement de glaucome [6, 11]. Harasymowycz et Coll. [12] ont publié une corrélation anatomo-clinique d´un patient avec ectropion de l'uvée et le glaucome néovasculaire sans anomalies systémiques. Le glaucome à la naissance associée à la NF1 peut être initialement diagnostiqué à tort comme le glaucome congénital unilatéral, en particulier chez les patients dont le neurofibrome plexiforme de la paupière et les taches café au lait ne sont pas évidents à un âge très jeune. Dans un autre sens, Chez les patients atteints de glaucome infantile unilatéral, il est conseillé de chercher des preuves de la NF1 et d'autres causes de glaucome unilatéral. L'examen des membres de la famille à la recherche des signes de NF1 et l´observation répétée pour le développement de neurofibrome plexiforme de la paupière, des taches café au lait, et des nodules de Lisch peuvent aider au diagnostic [10]. La gestion du glaucome congénital dans le cadre de la neurofibromatose est très complexe en raison de l'association fréquente de tumeurs orbitaires et palpébrales, de la dysplasie osseuse, et de son pronostic généralement médiocre à cause de plusieurs facteurs, y compris le décollement de la rétine, le gliome du nerf optique, l'amblyopie sévère causée par l'anisométropie, et la neuropathie optique glaucomateuse. Une prise en charge chirurgicale précoce permettrait à elle seule d'améliorer le pronostic.

## Conclusion

Nous soulignons à travers notre observation l'intérêt de la recherche précoce et systématique de glaucome congénital qui s'impose devant toute suspicion de neurofibromatose afin de réaliser une chirurgie précoce et adaptée pour pouvoir améliorer le pronostic.
